# aCNViewer: Comprehensive genome-wide visualization of absolute copy number and copy neutral variations

**DOI:** 10.1371/journal.pone.0189334

**Published:** 2017-12-19

**Authors:** Victor Renault, Jörg Tost, Fabien Pichon, Shu-Fang Wang-Renault, Eric Letouzé, Sandrine Imbeaud, Jessica Zucman-Rossi, Jean-François Deleuze, Alexandre How-Kit

**Affiliations:** 1 Laboratory for Bioinformatics, Fondation Jean Dausset–CEPH, Paris, France; 2 Laboratory of Excellence GenMed, Paris, France; 3 Laboratory for Epigenetics and Environment, Centre National de Recherche en Génomique Humaine, Institut de Biologie François Jacob, CEA, Evry, France; 4 Inserm, UMR-1162, Génomique fonctionnelle des tumeurs solides, Institut Universitaire d'Hématologie (IUH), Paris, France; 5 Laboratory for Genomics, Fondation Jean Dausset–CEPH, Paris, France; Universitat Zurich, SWITZERLAND

## Abstract

**Motivation:**

Copy number variations (CNV) include net gains or losses of part or whole chromosomal regions. They differ from copy neutral loss of heterozygosity (cn-LOH) events which do not induce any net change in the copy number and are often associated with uniparental disomy. These phenomena have long been reported to be associated with diseases and particularly in cancer. Losses/gains of genomic regions are often correlated with lower/higher gene expression. On the other hand, loss of heterozygosity (LOH) and cn-LOH are common events in cancer and may be associated with the loss of a functional tumor suppressor gene. Therefore, identifying recurrent CNV and cn-LOH events can be important as they may highlight common biological components and give insights into the development or mechanisms of a disease. However, no currently available tools allow a comprehensive whole-genome visualization of recurrent CNVs and cn-LOH in groups of samples providing absolute quantification of the aberrations leading to the loss of potentially important information.

**Results:**

To overcome these limitations, we developed aCNViewer (Absolute CNV Viewer), a visualization tool for absolute CNVs and cn-LOH across a group of samples. aCNViewer proposes three graphical representations: dendrograms, bi-dimensional heatmaps showing chromosomal regions sharing similar abnormality patterns, and quantitative stacked histograms facilitating the identification of recurrent absolute CNVs and cn-LOH. We illustrated aCNViewer using publically available hepatocellular carcinomas (HCCs) Affymetrix SNP Array data (Fig 1A). Regions 1q and 8q present a similar percentage of total gains but significantly different copy number gain categories (p-value of 0.0103 with a Fisher exact test), validated by another cohort of HCCs (p-value of 5.6e-7) (Fig 2B).

**Availability and implementation:**

aCNViewer is implemented in python and R and is available with a GNU GPLv3 license on GitHub https://github.com/FJD-CEPH/aCNViewer and Docker https://hub.docker.com/r/fjdceph/acnviewer/.

**Contact:**

aCNViewer@cephb.fr

## Introduction

Human cancers can be characterized by different levels of genomic instability. Whole or sections of chromosomes can be recurrently rearranged, lost, or amplified. Such changes can depend on either the type, the stage, or the grade of the tumors providing important clinical and biological information [3–5]. These gains/losses of genomic regions can have effects ranging from the simple gene dosage effect, where gains or losses are associated with a respectively higher or lower gene expression [6–8], to effects on chromatin structure with potential long-range effects on global expression [9]. In particular, LOH and cn-LOH events can be linked with a loss of function via the loss of a tumor suppressor gene as exemplified by the classical Knudson two-hit model [10,11]. These numerical or structural chromosomal abnormalities have been intensively characterized for nearly 50 years by increasingly resolutive methods. Earlier methods typically involved cytogenetics with (spectral) karyotyping [12]. This technique was succeeded by CGH [13] and SNP [14,15] arrays, and more recently next generation sequencing (NGS) [16,17]. CGH arrays only allow the identification of relative copy number variations [13] and many tools, such as CGHregions [18], CGHpro [19], have been developed for that purpose. On the other hand, SNP-arrays and NGS are more precise and enable the estimation of absolute copy number as well as copy neutral events. This became possible through the use of different developed algorithms such as PICNIC [20], GAP [21], ASCAT [22], OncoSNP [23], GPHMM [24], ExomeCNV [25], VarScan2 [26], ABSOLUTE [27] and Sequenza [28]. Some of these tools [22,28] can also predict the average ploidy and/or the percentage of contamination of the tumor with constitutional DNA from normal cells. CGH arrays produce log2 intensity ratios, LRR, between the samples of interest and a reference sample allowing the identification of regions with gains (LRR > 0) or losses (LRR < 0). From SNP array and WGS/WES data though, it is possible to calculate LRR values along with B Allele Frequencies (BAF) given by the formula: b / (a+b) with a and b being the intensities of the a and b alleles respectively. The addition of BAF values to LRR values facilitates absolute copy number estimates. Indeed, both ASCAT and Sequenza introduce Ψ, the sample ploidy, and ρ, the percentage of tumor cells within the sample of interest along with n_a_ and n_b_, the number of copies of alleles a and b in their respective models. ASCAT will use two equations (one for LRR and one for BAF) expressed as functions of the unknowns, Ψ, ρ, n_a_ and n_b_. By rearranging these two equations, n_a_ and n_b_ will then be expressed as functions of the LRR and BAF values, Ψ, and ρ. By iterating through a range of realistic values for Ψ and ρ, different values of n_a_ and n_b_ are calculated along with a goodness-of-fit score in order to estimate n_a_, n_b_, Ψ, and ρ. Conversely, Sequenza uses a probabilistic model where the probability densities of LRR and BAF are modeled using a non-standardized Student's t-distribution. Sequenza uses a maximum a posteriori approach over a range of realistic values for Ψ and ρ to first estimate Ψ and ρ before estimating n_a_ and n_b_. These algorithms treat each sample individually and display different output files which show copy number or copy neutral variations and tumor ploidy and/or the percentage of tumoral DNA in the samples. However, they do not provide a genome-wide visualization of the different chromosomal aberrations for groups of samples; this would facilitate the identification of recurrent events. Other tools have been developed for the visualizations of CNVs of different samples simultaneously in one figure. These either use CGH array data such as in ChARMView [29], or SNP array or/and NGS data such as in Circos [30], YMAP [31], the extensively used Integrative Genomics Viewer [32] and the commercially available Nexus Copy Number^TM^ Software (Biodiscovery). However, these visualization tools only represent the chromosomal aberrations in a relative manner. Only gains and high gains or losses and high losses are indicated even when the algorithm used for the CNV analysis gives absolute CNV results. Hence information is lost when using these tools. Furthermore, some of them do not allow a representation of all the samples in one superimposed figure where frequencies of the events can be visually identified [29] or they show only a chromosomal view instead of a whole genome view [32].

Here, we developed aCNViewer, a genome-wide visualization tool for the representation of absolute copy number and copy neutral variations (chromosome losses and gains, LOH and cn-LOH) of groups of samples. This includes a whole pipeline for processing raw SNP and whole exome/genome paired (tumor/peritumoral) bam data. aCNViewer allows the easy identification of recurrent events through three different graphical outputs: dendrograms, bi-dimensional heatmaps, and stacked histograms. These represent a comprehensive visualization of copy number and copy neutral variations which might help the understanding of the underlying biology of the tumors and identify potential candidate genes implicated in tumorigenesis.

## Results & discussion

**Fig 1:**
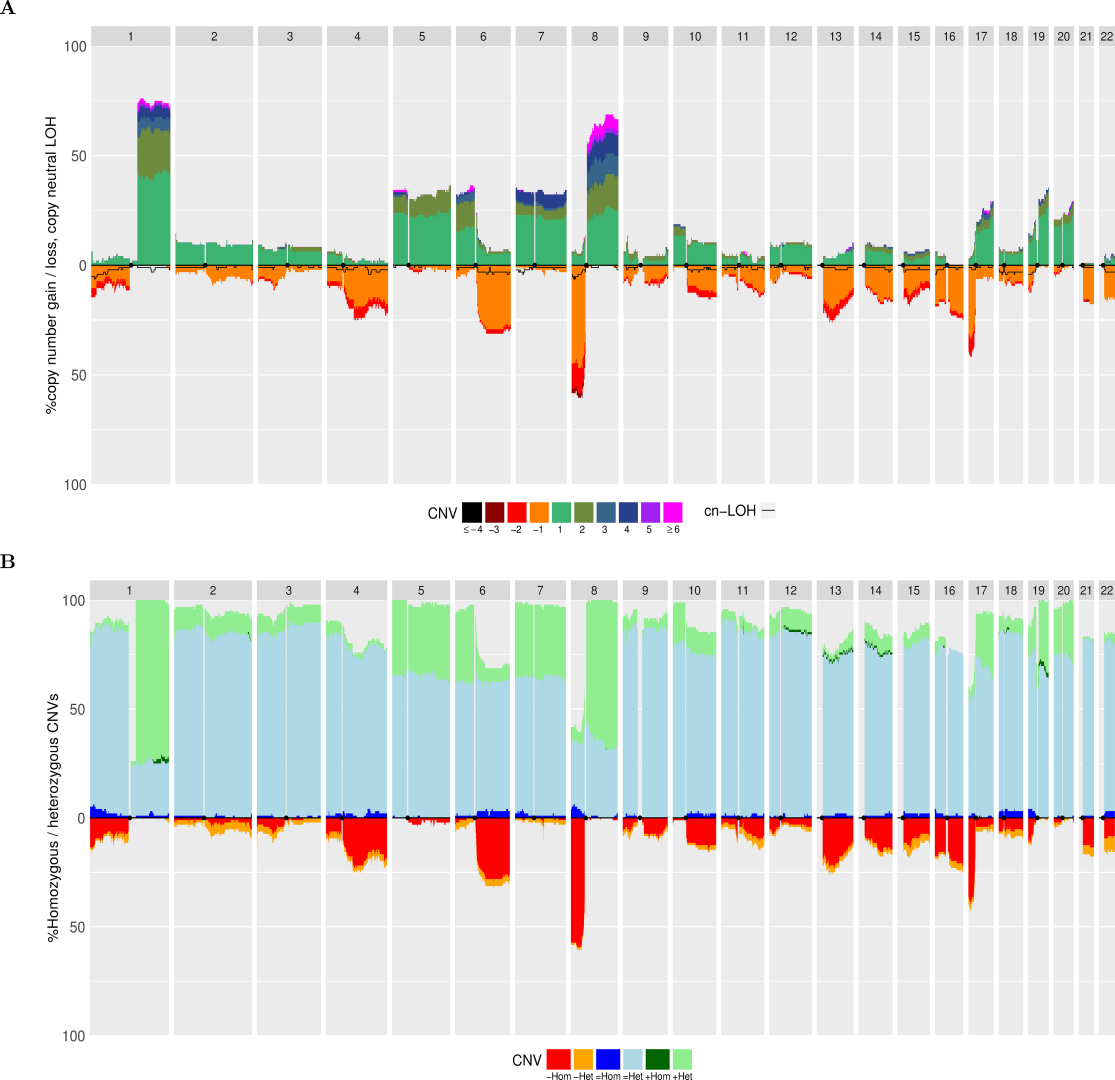
Quantitative stacked histograms using 96 HCC samples on Affymetrix 500K Human Mapping Array data from [1]. A) Frequency of CNV and cn-LOH events along the genome. The left axis indicates the frequency of gains or losses among the 96 samples and the legend below indicates the number of copy number gains or losses from the reference baseline. The black line indicates the frequency of cn-LOH along the genome in negative ordinates. B) Frequency of homozygous/heterozygous CNVs along the genome. Copy-neutral events / gains and losses are respectively displayed in positive and negative ordinates.

aCNViewer includes a whole pipeline for processing raw SNP and whole exome/genome paired (tumor/peritumoral) bam data with ASCAT [22] and Sequenza [28] algorithms respectively which allow the identification of absolute copy number and copy neutral variations for each sample individually (Fig 3). The CNV data are converted into a matrix using a basic windowing approach. The user specified window length gives the resolution used by aCNViewer to compute dendrograms and bi-dimensional heatmaps. Additionally, from the CNV data, genomic segments with associated sample names and copy number values are merged to obtain non-overlapping segments containing the list of all samples sharing a related genomic position and copy number value. The estimated ploidy for each sample is subtracted from the copy number values of these resulting segments or, alternatively, from the entries of the matrix constructed for dendrograms and heatmaps. Thus, a copy number of 0 means no copy number change. These adjusted windows or the matrix of segments at base resolution (see section “Construction of M_s_, a matrix of segments at base resolution” below) are then plotted into a stacked histogram representing genome-wide absolute copy number and copy neutral variations over all samples in a group (Fig 1A). aCNViewer also outputs a record of recurrently aberrant regions with the frequency of each event along with different statistics implemented in GISTIC [33].

**Fig 2:**
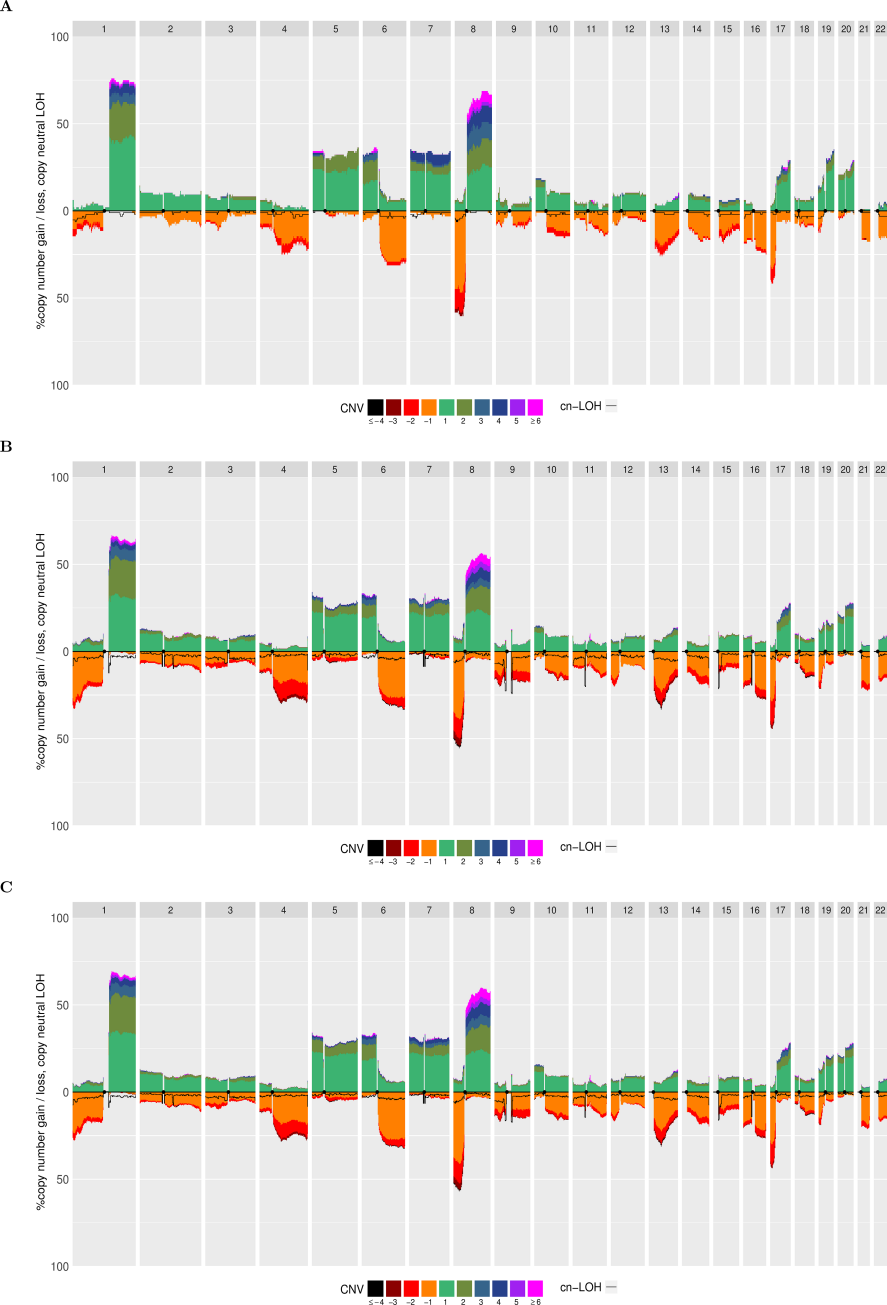
Quantitative stacked histograms produced by aCNViewer showing the frequency of CNVs and cn-LOH along the genome in HCCs. Quantitative stacked histograms generated using A) 96 freely available HCC Affymetrix 500K Human Mapping Array data [1], B) 243 HCC WES experiments from [2] and C) 317 pooled HCCs from both SNP and WES experiment data.

In order to illustrate aCNViewer, we processed publically available Affymetrix 500K Human Mapping Array data on 96 hepatocellular carcinomas (HCCs) [1] classified according to Barcelona Clinic Liver Cancer (BCLC) staging and generated the graphical outputs using 2Mb windows. The dendrogram allows the identification of two main groups of HCCs based on CNVs. These did not cluster according to their BCLC staging (Fig 4A) nor other clinical or molecular features available. In the bi-dimensional heatmap representation of CNVs, the absolute CNVs (0–8 copies and >8) are shown in all HCC samples and reveal that the samples cluster in two main groups according to their ploidy: 1) diploid and pseudo-diploid tumors and 2) polyploid (mainly pseudo-tetraploid) tumors (Fig 4B). Finally, the stacked histogram (Fig 1A) allowed the easy detection of recurrent CNVs and cn-LOH among HCCs. The zero baseline indicates the “normal” copy number of the chromosomes, which is in most cases diploid but can also be triploid, tetraploid or more. The use of a correct baseline is essential for the identification of recurrent CNVs as exemplified in S1 Fig where one pseudo-diploid HCC sample and one pseudo-tetraploid HCC sample harbored most of the shared chromosomal aberrations (chr 1q gain, chr 8p loss, and chr 8q gain). Differences between the two tumors would not have been revealed in the pseudo-tetraploid HCC sample if the ploidy baseline of both samples were set by default to 2. All reported recurrent gains and losses listed in Fig 1 of [1] are also found in our histogram (Fig 1A). Moreover, due to the representation of absolute CNVs, statistically significant differences (p-value of 0.0103 with a Fisher exact test) between the two most frequent CN gains can be found: region 1q presents a copy gain “≥ +4” for about 7% of the samples compared to 25% of the samples for region 8q (Fig 1A). These results were confirmed in another independent set of 243 HCC samples from WES experiments [2]. aCNViewer was used to reproduce the differences in CN gains between regions 1q and 8q (S2B Fig, p-value of 5.6e-7 with a Fisher exact test). This suggests a possible biological implication of these quantitative CNV differences in HCC and confirms the need of such a representation of CNV and cn-LOH for a better understanding of the underlying biology of the disease (losses seem to be more pronounced, though, in the histogram generated using the WES data (Fig 2B) compared to the one from the SNP array data (Fig 2A). See Supplementary section “Comparison of the quantitative stacked histograms between SNP array and WES data” in S1 File for more details). By using CGHregions [18] on the Affymetrix data set [1], we confirmed that the global trend of gains and losses are consistent with ASCAT results (S2 Fig) (some differences are notable though and this is discussed in the supplementary section “Comparison of the quantitative stacked histograms using SNP array data from [1] processed with ASCAT and CGHregions” in S1 File). Finally, we merged HCC CNV data from Affymetrix 500K Human Mapping Array and WES sequencing experiments processed with ASCAT and Sequenza respectively to obtain a whole-genome visualization of recurrent CNV and cn-LOH events on a larger group of samples. Thus, we demonstrate the possibility by aCNViewer to generate a single graph based on data from multiple experimental designs in order to gain more statistical power (Fig 2C). 1,237 samples from Hapmap3 on the Affy6 platform (ftp://ftp.ncbi.nlm.nih.gov/hapmap/raw_data/hapmap3_affy6.0/) were processed using aCNViewer (S5 and S6 Figs). As expected, few recurrent copy number events were present.

**Fig 3:**
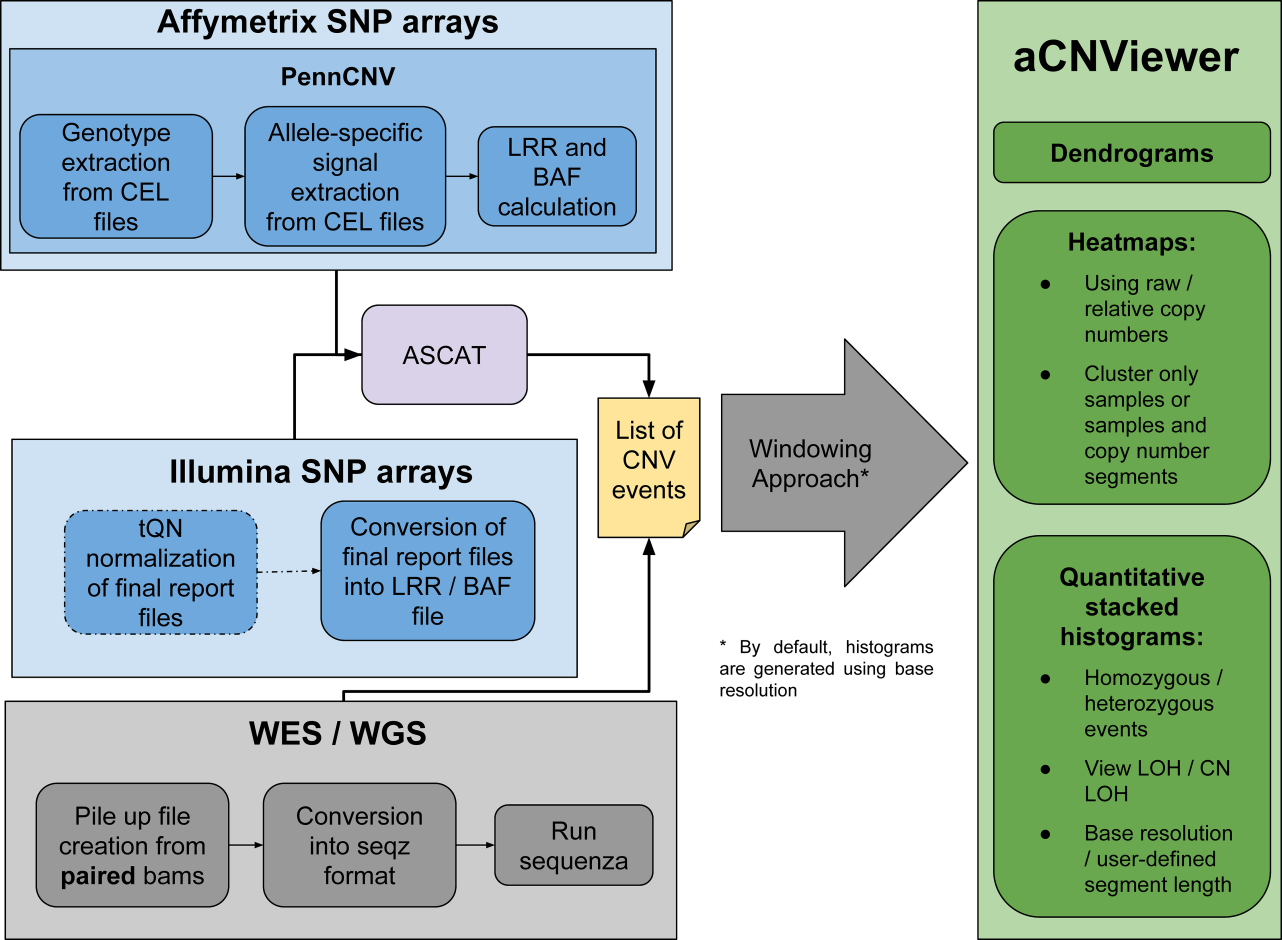
Overview of the different steps handled by aCNViewer. aCNviewer can process Affymetrix and Illumina SNP arrays as well as NGS data. LRR and BAF files are obtained after processing SNP raw data by PennCNV for Affymetrix and a threshold quantile normalization (tQN) for Illumina and subsequent use of ASCAT for CNV and cn-LOH detection. For NGS data, paired tumoral and non-tumoral whole exome/genome sequencing bam data are converted into seqz format and processed by Sequenza for CNV detection. aCNViewer converts CNV data into a CNV matrix with the window size defined by the user and which is subsequently used to compute dendrograms and heatmaps. Quantitative stacked histograms can be generated using the same matrix or a matrix of segments at base resolution (default behaviour). Text files are also available through GISTIC [33] providing a robust statistical way to select recurrent CNVs.

**Fig 4:**
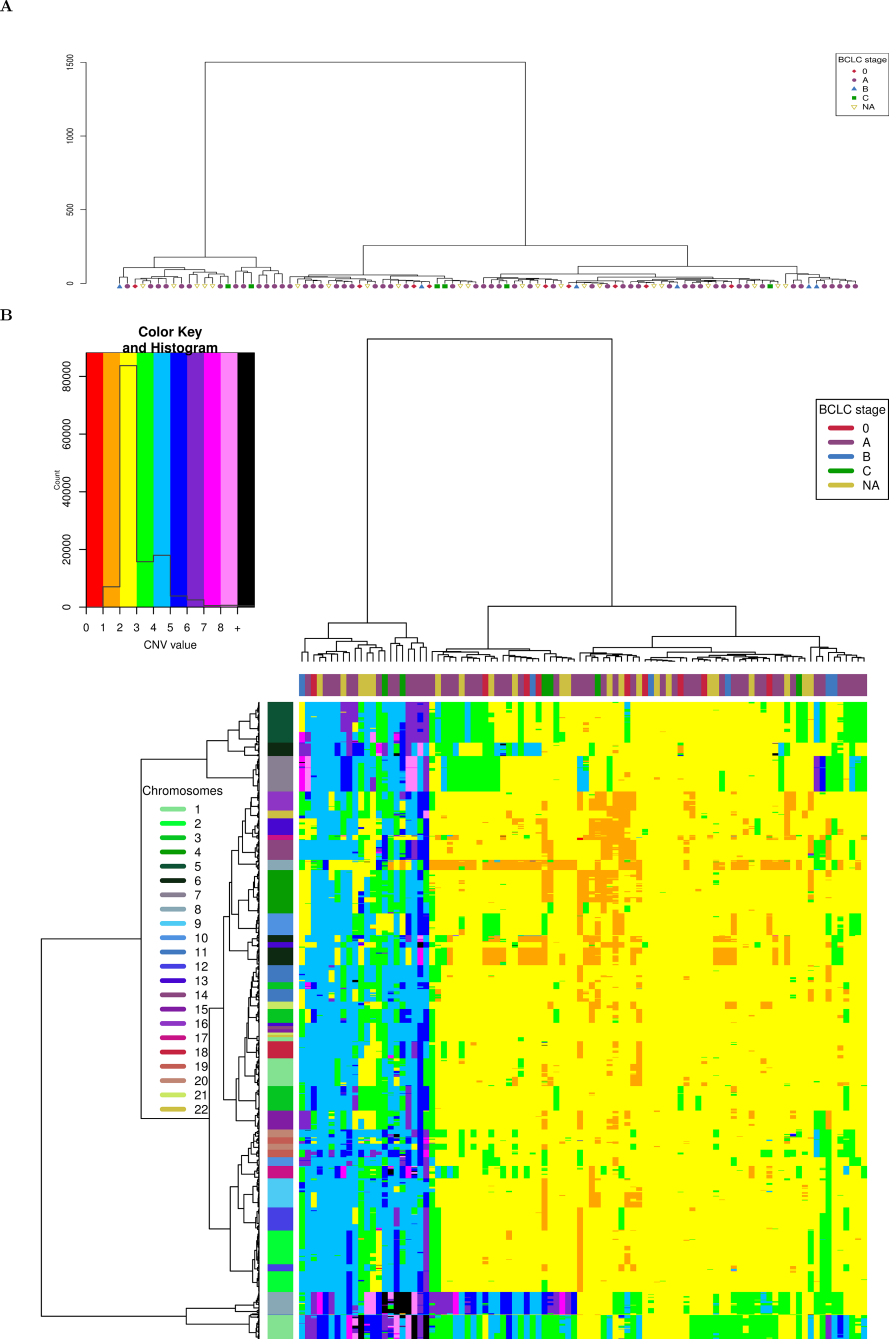
Hierarchical clustering of HCCs from [1] according to BCLC staging and based on CNVs. A) Dendrogram representation. B) Bi-dimensional heatmap. A 2Mb window length is used for computation. The chromosomes of each window are shown on the right and the BCLC staging of each tumor is given on top of the bi-dimensional heatmap.

aCNViewer has certain limitations including the fact that it does not currently account for intra-tumor heterogeneity. Having a simultaneous view of the copy number landscape along with the clonality status of these events could help to better understand the mechanisms of the disease [34]. Another current limitation of aCNViewer is the absence of a function to compare two groups of samples. One simple way to do that, though, would be to generate the quantitative histograms for both groups separately and compare these plots as we did in Fig 2.

## Design and implementation

aCNViewer relies on the absolute copy number and copy neutral variations obtained with ASCAT and Sequenza which can respectively process SNP arrays and NGS data (Fig 3). ASCAT and Sequenza results go into a basic windowing approach according to a user-defined window length or chromosomal fraction. This determines the resolution of the representation, generating a matrix of copy number or copy neutral events (Fig 3). This matrix (or alternatively a matrix of relative copy number values obtained by subtracting the associated estimated sample ploidy from each copy number value) is then used to compute firstly dendrograms for the identification of samples sharing the same chromosomal aberrations (Fig 4A) and secondly bi-dimensional heatmaps (Fig 4B) for the identification of chromosomal regions with similar abnormality patterns. Finally, using either the matrix of relative copy number values or a matrix of segments at base resolution (see section “Construction of M_s_, a matrix of segments at base resolution” below), a quantitative stacked histogram is generated with absolute whole genome copy number and copy neutral variation profiles showing the frequency of recurrent events across all selected samples (Fig 1A). A similar histogram is generated with a whole genome view of homozygous/heterozygous CNVs (Fig 1B). aCNViewer is implemented in python for the pipelining steps and in R for the generation of dendrograms, heatmaps, and stacked histograms and thus runs on all linux/unix platforms.

Let us define C = {C_A_(i)} as the list of copy number segments extracted from ASCAT / Sequenza or any other CNV caller chosen by the user for all samples A and genomic positions i. In some instances, C_A_(i) will be simply named C when the reference to a specific sample and genomic position is unnecessary.

### Generation of M, a matrix of copy number values

Each chromosome is divided into non-overlapping windows of a user-defined length, L, specified either in number of bases or percentage of chromosome length. For each window W and for each sample A, all CNV data in that interval is retrieved and potential missing regions (for SNP arrays, ASCAT may not give a CNV value between two sets of probes with distinct CNV values and for NGS, Sequenza will not assign any value for uncovered regions) are inserted into the CNV data with A’s sample ploidy, calculated as described in the section “Ploidy calculation” below, so that each base in W has a CNV value. The global CNV value of W for sample A is then set to be the average of the CNV values within W rounded to the nearest integer. There is one particular case: if W is overlapping with a centromeric region E, W becomes W—E and if the new length of W < L/2, W is merged with its nearest contiguous window W’ in order to obtain a window of comparable size to the others.

After this step, a matrix M of CNV values with samples as rows and windows as columns is constructed and will be the basis for most of the subsequent plots.

#### Ploidy calculation

A matrix M_p_ of CNV values is constructed using a resolution of 10% of the chromosome length and a default ploidy of 2 for “missing” CNV values (as the window length is relatively large, the default ploidy will actually not have a great effect on the CNV value of each window). From M_p_, for each sample A, all the windows related to A and their related CNV values, C_A_ = {C_A_(i)} for all genomic positions i, are extracted. The ploidy of A is set to be the most frequent CNV value in C_A_. If several values appear with the same frequency, the ploidy is set to the average of these values. Our ploidy estimates had 95% concordance with ASCAT tumor ploidy estimates rounded to an integer. In order to have a consistent way to evaluate the sample ploidy for SNP arrays and WGS/WES data, our method is used by default. It is, however, customizable and the user can choose to use ASCAT, Sequenza (set *useCustomPloidies* to 1), or user-defined ploidies.

#### Generation of a matrix of relative copy number values

Copy number values are calculated by ASCAT or Sequenza if aCNViewer is used on SNP array or raw sequencing data. Alternatively, they can be calculated from other tools as long as they are provided in the ASCAT segment format (https://github.com/FJD-CEPH/aCNViewer#othercnvformats). By default, these copy number values are used by aCNViewer to produce dendrograms and heatmaps. Relative copy number values can also be used (set option *useRelativeCopyNbForClustering* to 1) to produce the same types of graphical representations. In this case, for each sample A, the relative copy number values C’_A_ are calculated using the following formula for all genomic positions i: C’_A_(i) = C_A_(i)—p_A_ where C_A_(i) is the copy number value for genomic position i and sample A and p_A_ is the estimated ploidy of sample A. Thus, C’_A_(i) is 0 when the copy number value is equal to p_A_ and it is negative when the copy number value is lower than p_A_. C’_A_ (i) has a minimal value of -p_A_ in the case where C_A_(i) is 0 but it has no upper limit. Thus a respective copy number value of -3 and -4 is possible only for samples with p_A_ ≥ 3 and p_A_ ≥ 4 respectively. In order to keep the graphical representations readable, we limited the values of C’_A_(i) so that -4 ≤ C’_A_(i) ≤ 6. Using the matrix M, a matrix of relative copy number values M’ can be constructed using the definition of C’_A_(i).

### Dendrograms

Using M or M’ as an input, R’s hclust function is used to generate dendrograms with the default agglomeration method set to “ward.D”; though this setting can be changed by the user. The plot of the dendrograms has been customized to show shaped colored leaves representing all the different groups each sample belongs to. aCNViewer can also generate a set of dendrograms for each feature listed in a file with clinical information. This allows an easy visual inspection in order to spot the main features correlated with the dendrogram structure (Table 1).

**Table 1:** aCNViewer main options.

Category	Option (default value)	Description
**General**	—plotAll (1)	specify whether all available plots should be generated (values are 0 or 1)
	—refBuild REF_BUILD	the genome build used to generate the CNV segments (hg18 and hg19 are currently supported. For custom build, please check the github website°
	-w WINDOW_SIZE (2000000) / -p PERCENT	WINDOW_SIZE defines the window length in bp used to cut the genome in order to generate a matrix of CNV events. Alternatively, PERCENT can be used instead of WINDOW_SIZE in order to set the window size in percentage of chromosome length where PERCENT is a floating number between 0 and 100.
	-t TARGET_DIR	set the path of the output folder
	-b BIN_DIR	set the path of the folder containing all required binaries. For a detailed description of the structure, please refer to https://github.com/FJD-CEPH/aCNViewer#binDir.
	-f FILE_NAME	Path to the CNV file in PennCNV/ASCAT format. Can also process Sequenza results and in that case the following option—fileType Sequenza should be added and FILE_NAME should point to the folder containing Sequenza results.
	—ploidyFile FILE_NAME /—useCustomPloidies USE_CUSTOM_PLOIDIES (1)	Can either be a tab-delimited file with at least 2 columns: "sample" and "ploidy" or an integer, which will set the same ploidy to all samples. By default (USE_CUSTOM_PLOIDIES is 1), the ploidy is calculated using the CNV file grouped into windows of 10% of chromosomal length. The ploidy is then set to be the most represented CNV value for each sample. It is possible to use ASCAT/Sequenza ploidies by leaving FILE_NAME to null and by setting USE_CUSTOM_PLOIDIES to 0.
	—runGISTIC (0)	specify whether to run GISTIC in order to have a statistical way to prioritize regions of interest (values are 0 or 1)
	—smallMem SMALL_MEM (0)	If small_mem is 1, GISTIC will run in small memory mode and will only require about 10GB of RAM vs 50GB of RAM otherwise at the expense of a longer running time.
	—rColorFile FILE_NAME	file* allowing to customize graph colors
	—outputFormat FORMAT	allow to customize output formats for the different types of available plots (histograms, heatmaps and dendrograms). The default value is hist:png(width = 4000,height = 1800,res = 300);hetHom:png(width = 4000,height = 1800,res = 300);dend:png(width = 4000,height = 2200,res = 300);heat:pdf(width = 10,height = 12). For more information, please refer to https://github.com/FJD-CEPH/aCNViewer#outputFormat.
**histogram**	—lohToPlot LOH_TO_PLOT (cn-LOH)	Tell what values should be added to the histogram. Values should be one of "cn-LOH" for plotting cn-LOH only, "LOH" for LOH only, "both" for cn-LOH and LOH or "none" to disable this feature.
	—useFullResolutionForHist (1)	tell whether to plot histogram using full (base) resolution i.e. CNVs are not grouped into windows according to a user-defined length. If 0, the resolution of the plot will be given by either WINDOW_SIZE (option -w) or PERCENT (option -p)
**Heatmap**	—useRelativeCopyNbForClustering (0)	indicate whether the CNV matrix used for the heatmap should be relative copy number values or raw copy number
	—keepGenomicPosForHistogram (0)	if set to 1, the fragmented genome is kept in its original position and not cluster windows according to sample CNV patterns
**Heatmap/dendrogram**	—sampleFile SAMPLE_FILE	a tab-delimited file that should contain a column named Sample with the name of each sample and at least another column with the phenotypic/clinical feature. This file can contain a sample alias, which will be used as the official sample id if provided. This parameter can be used for dendrograms as well.
	-G FEATURE_NAME	refers to the name of the column of the phenotypic/clinical feature of interest in SAMPLE_FILE if specified. If you omit this parameter, one plot per feature defined in SAMPLE_FILE will be generated. This file can contain a sample alias, which will be used as the official sample id if provided. This parameter can be used for dendrograms as well.

* an example can be found at https://github.com/FJD-CEPH/aCNViewer/blob/master/img/rColor.txt

° for more information, please check the github website: https://github.com/FJD-CEPH/aCNViewer

### Heatmaps

Using M or M’ as an input, R’s heatmap.2 function in the *gplots* package is used in aCNViewer with the default hierarchical clustering function set to *hclust* and the default agglomeration method set to “ward.D” (both parameters are customizable by the user). Similarly to dendrograms, it is possible to generate one heatmap for each feature listed in a file with clinical information (see option “sampleFile” in Table 1). The other options for bi-dimensional heatmap include the possibility to cluster the chromosomal windows (S3A Fig) and the possibility to use the absolute CNV data, the absolute CNV data relative to the estimated tumor ploidies (S3B Fig), or relative to a standard ploidy of two (S3C Fig, Table 1).

### Stacked histograms

Stacked histograms allow the representation and identification of recurrent CNV and cn-LOH events along the genome in groups of samples while retaining the quantitative information present in CNV data. This has never been performed before. Default stacked histograms will be generated using the matrix of segments at base resolution M_s_ obtained from raw copy number data or alternatively M’.

#### Construction of M_s_, a matrix of segments at base resolution

Let us define M_s_ = {K_A_(i)} as the matrix of segments at base resolution for all samples A and genomic positions i. M_s_ will be constructed from C simply by segmenting the genomic positions i so that they are all non-overlapping. This is equivalent to having the windowing approach, described in section “Generation of M, a matrix of copy number values”, with the advantage of not having to fill in “the blanks” for a given window. First, C is sorted by genomic position. Then, we iterate through C in order to create the segments K_A_(i) as follows: first consider two consecutive copy number segments C_A_(i) and C_B_(j) for respectively sample A at genomic position i and sample B at genomic position j. Note that A equals B only if the genomic positions i and j are non-overlapping (i.e. a CNV caller will produce non-overlapping CNV calls for any given sample). If A equals B then we define K_A_(i) = C_A_(i) and K_B_(j) = C_B_(j). If i and j are overlapping (and thus A ≠ B), we have, at most, three sets of genomic positions to consider: i∩j (the intersection of i and j), i-j (the genomic position present in i but not in j) and j-i. We instantiate K for these genomic locations as follows: K_A_(i∩j) = C_A_(i), K_B_(i∩j) = C_B_(j), K_B_(j-i) = C_B_(j) and K_A_(i-j) = C_A_(i).

After going through all the segments in C, we obtain the matrix M_s_ of CNV values with samples as rows and segments (with various lengths but all non-overlapping) as columns.

#### Histogram creation

M_s_ (default value) or M’ is used as an input and for each segment S (or window for M’) and for each copy number value C (ranging from -4 to +6), the percentage of overall samples in S having the copy number value C can now be calculated. These percentages in S for each copy number value C are then stacked and plotted according to the genomic position of S in positive ordinates for gains and in negative ordinates for losses.

Cn-LOH/LOH calculation: ASCAT and Sequenza generate allele-specific CNVs and thus allow the identifications of cn-LOH by considering only copy number events where one of the two alleles has no copies and the other one has the same number of copies as the sample ploidy. By considering only these events, we can generate the matrices cn-M’ and cn-M_s_ equivalent to their counterparts M’ and M_s_. For each event in cn-M’ or cn-M_s_, the total percentage of samples having this event is calculated. The copy neutral variations are then plotted in the stacked histograms as a black line indicating for each segment or window the percentage of samples presenting the current cn-LOH in negative ordinates.

Stacked histograms options include the possibility to plot LOH represented as a blue line indicating the percentage of samples presenting LOH in negative ordinates (see option “*lohToPlot”* in Table 1, S4A Fig). The estimated sample ploidy is used by default (S4A Fig) or can be adjusted using a user defined value (see option “ploidyFile” in Table 1, S4B Fig with a ploidy of two for every sample).

The stacked histograms are also represented in text format with the list and percentage of samples for each copy number value C allowing the user to easily identify samples of interest (Fig 1A). GISTIC results with focal and broad copy number events with their associated statistics are also available if the “*runGISTIC”* option has been enabled giving the user the choice of the criteria for selecting potentially interesting events.

#### Heterozygous/homozygous CNVs

Similarly to the construction of cn-M’ and cn-M_s_, we can construct hH-M’ and hH-M_s_ by considering raw CNV data as segments with the following features: sample name, gain, no net change of copy number or loss and a status indicating whether the segment is heterozygous (both alleles have non null copy number values) or homozygous (at least one the alleles has a null copy number value). By adding for each event in hH-M’ or hH-M_s_ the percentage of samples presenting the event, a stacked histogram can be plotted with either gains or no net change of ploidy (the copy number value is equal to the sample ploidy) represented in positive ordinates and losses in negative ordinates (Fig 1B).

### Output files and options

For each processed sample, all the output files produced by ASCAT and Sequenza are fully available and listed in Table A in S1 File. The resulting CNV data are then used as an input data in aCNViewer. The user may manually exclude samples from the analysis if desired (see options “sampleToExcludeList” and “sampleToProcessList” in Table 1) and the automated ploidy estimation of each sample can also be modified at the user’s convenience (see “ploidyFile” option in Table 1). The user can define different groups of samples according to any characteristics (age groups, gender, tumor stage, etc.) to be processed by aCNViewer (see “sampleFile” option in Table 1).

aCNViewer allows three types of high quality graphical outputs suitable for publication: dendrograms, bi-dimensional heatmaps, and stacked histograms in jpg/png/tiff/bmp/pdf format whose resolution is defined by the user (see “outputFormat” option in Table 1). The colors used in the graphs are set by default but are totally customisable (see option “rColorFile” in Table 1). Moreover, it also produces text format files allowing the easy identification of samples with recurrent CNV events using GISTIC [33].

## Availability and future directions

Following the accelerating global trend towards precision medicine, there is an increased need for evermore precise tools to help physicians gain insights from the rapidly accumulating available data. Having access to clear and precise pictures summarizing CNVs and cn-LOH genome-wide could help to achieve more comprehensive interpretations. We have shown that aCNViewer can help identifying rapidly recurrent CNVs in datasets from Affymetrix SNP arrays as well as WES/WGS data. The application and source code are available as open source on GitHub and Docker and a demo can be found at https://github.com/FJD-CEPH/aCNViewer. Future developments of aCNViewer include the adaptation of the quantitative histograms and statistics on methylation data where copy number events would be replaced by variations in methylation levels. To our knowledge, this type of representation on methylation data would be original and would allow the rapid identification of group of samples sharing the same methylation pattern in specific regions of the genome. This could be further extended to any type of data by considering a matrix of event densities where each value of this matrix would represent the density of a given event (reads, single nucleotide variants, indels, somatic variants, etc.) in each genomic window. This representation would help identify regions with a high density of a given event shared by a large number of samples.

## Supporting information

S1 File This file contains supplementary sections “Comparison of the quantitative stacked histograms between SNP array and WES data”, “Comparison of the quantitative stacked histograms using SNP array data from [1] processed with ASCAT and CGHregions” and Table A (List of files produced by ASCAT and Sequenza). (DOCX)

S1 Fig ASCAT profile of two HCCs including a pseudo-diploid sample (A) and a pseudo-tetraploid sample (B) and presenting similar chromosomal aberrations.
(TIF)

S2 Fig **Quantitative stacked histograms produced by aCNViewer showing the frequency of CNVs and cn-LOH along the genome in HCCs using** 96 freely available HCC Affymetrix 500K Human Mapping Array data [1] **processed by ASCAT (A) and CGHregions (B).**
(TIF)

S3 Fig Various options for bi-dimensional heatmap graphical representations of HCCs from [1] using a window length of 2Mb. Bi-dimensional heatmap representations without clustering of chromosomal windows with (A) absolute CNV data, (B) absolute CNV data relative to the estimate tumor ploidy and (C) absolute CNV data relative to a ploidy of 2.
(TIF)

S4 Fig Quantitative stacked histograms generated on data from [1] showing the importance of the choice for each sample’s ploidy. A) The estimated sample ploidy has been taken into account to generate relative copy number values. The blue line represents all LOH events. B) Using a ploidy of 2, cn-LOH events are represented by the black line.
(TIF)

S5 Fig Quantitative stacked histogram on Affy6 Hapmap3 data. (TIF)

S6 Fig Heatmap on Affy6 Hapmap3 data using 2Mb windows. (TIF)
