# Supplementary material for: aCNViewer: Comprehensive genome-wide visualization of absolute copy number and copy neutral variations
Source: PLoS One. 2017 Dec 19;12(12):e0189334. doi: 10.1371/journal.pone.0189334 (PMC5736239; doi:10.1371/journal.pone.0189334)
Supplement: S1 File — (DOCX) [file pone.0189334.s001.docx]

**Supporting Information**

**Comparison of the quantitative stacked histograms between SNP array and WES data**

The quantitative stacked histograms generated using SNP array (Fig 2A) and WES data (Fig 2B) are very similar in terms of copy number gain/loss patterns. Losses seem to be more pronounced in the WES data though. Several factors could explain this fact. The first obvious difference between these data is that the SNP array data has been analyzed using ASCAT in tumor only mode while the WES data has been analyzed using Sequenza in paired (T/NT) mode. Thus, one possible explanation for the observed differences could be that ASCAT results are less precise as only tumor data was used. Another possibility could simply be the differences in sample size and sample composition between the two cohorts: the SNP array sample cohort contains 96 samples out of which 77.1% are diploid compared to 243 samples with 74.1% diploid samples in the WES cohort. Looking closely into the regions where differences in loss level between the WES and SNP array data happen (regions 4q, 6q, 8p, 13q and 18p and q), we can see that it is mostly caused by the presence of one sample in the WES cohort (not necessarily the same in each region) having a relative copy number value of -3 or -4.

**Comparison of the quantitative stacked histograms using SNP array data from** [**[1]**](https://paperpile.com/c/AyOaNl/kNzX) **processed with ASCAT and CGHregions**

S2B Fig shows the plot produced using CGHregions segments and we can clearly see that peaks and positions match pretty well between ASCAT (S2A Fig) and CGHregions. Putting aside the loss of information due to CGHregions not supporting absolute copy number values, we do see some slight differences in terms of peak heights with ASCAT peaks reported to be higher compared to CGHregions: for example, for arm 1q about 65% of samples show an amplification for CGHregions while about 75% is reported in ASCAT. These differences are, most likely, attributable to the dimension reduction implemented in CGHregions where a representative region will be calculated using the medoid of all clones in that region. The other noticeable differences between both histograms are the presence of focal events in the plot of CGHregions while these events are absent from ASCAT (and also absent from the plot obtained using SEQUENZA in Fig 2B). We chose 3 genomic locations showing a steep peak in CGHregions and absent from ASCAT results for further investigations: losses in 5q and 21q regions (position 5:162054724-162524106, 21:38200172-38720023) and the gain in 18q (position 18:37437085-37437400). For each of these regions, we identified the samples that were involved in the reported gains or losses. We went back to the log R ratios and found, indeed, evidence supporting respectively losses and gains for respectively regions 5q and 21q and 18q. In order to call CNVs, ASCAT is using only SNPs that are heterozygous in the germline samples. In tumor only mode, ASCAT is predicting germline genotypes by assuming homozygous sites in tumors are also homozygous in the germline. Looking closely at these 3 regions, more than half of the SNPs available in these regions are skipped by ASCAT as they are predicted to be homozygous in the germline. In the next step, ASCAT uses the Allele-Specific Piecewise Constant Fitting algorithm on the remaining SNP data in order to find an optimal partitioning of consecutive probes into segments. This last step is combining the remaining SNP data in the 3 regions with many other surrounding SNPs resulting in a smoothing which removes these 3 peaks from ASCAT results. It could be argued that these peaks are not noise but true signals and that’s why we give the user the possibility to give any other CNV data as an input to aCNViewer (<https://github.com/FJD-CEPH/aCNViewer#othercnvformats>). In conclusion, results from ASCAT and CGHregions are comparable modulo the differences induced by the intrinsic differences of algorithms.

**Table A. List of files produced by ASCAT and Sequenza**

| **ASCAT files** | **Description** |
| --- | --- |
| .ASCATprofile.png | genome-wide representation of ASCAT CNVs |
| .ASPCF.png | results of segmentation using Allele-Specific Piecewise Constant Fitting |
| .rawprofile.png | genome-wide representation of raw ASCAT CNVs |
| .sunrise.png | sunrise plot showing the optimal solution of tumor ploidy and percentage of aberrant tumor |
| .tumour.png | representation of LogR and BAF values |
| tumorSep*.png | plot of BAF values |
| .ascatInfo.txt | ASCAT values of aberrant cell fraction, goodness of fit, psi and ploidy for all samples |
| .segments.txt | list of all CNVs with the copy number for each allele |
|  |  |
|  |  |
| **Sequenza files** | **Description** |
| *_segments.txt | predicted CNVs |
| *_CP_contours.pdf, *_confints_CP.txt & *_model_fit.pdf | inferred cellularity and ploidy |
| *_alternative_fit.pdf & *_alternative_solutions.txt | alternative inferred cellularities and ploidies |
| *_chromosome_view.pdf | chromosome view with mutations, BAF, depth ratio, and segments |
| *_genome_view.pdf | genome view of all the CNVs |
| *_mutations.txt | list of detected mutations |
| *_CN_bars.pdf | frequency of all the copy number values |
