# Supplementary figures and images for: aCNViewer: Comprehensive genome-wide visualization of absolute copy number and copy neutral variations
Source: PLoS One. 2017 Dec 19;12(12):e0189334. doi: 10.1371/journal.pone.0189334 (PMC5736239; doi:10.1371/journal.pone.0189334)

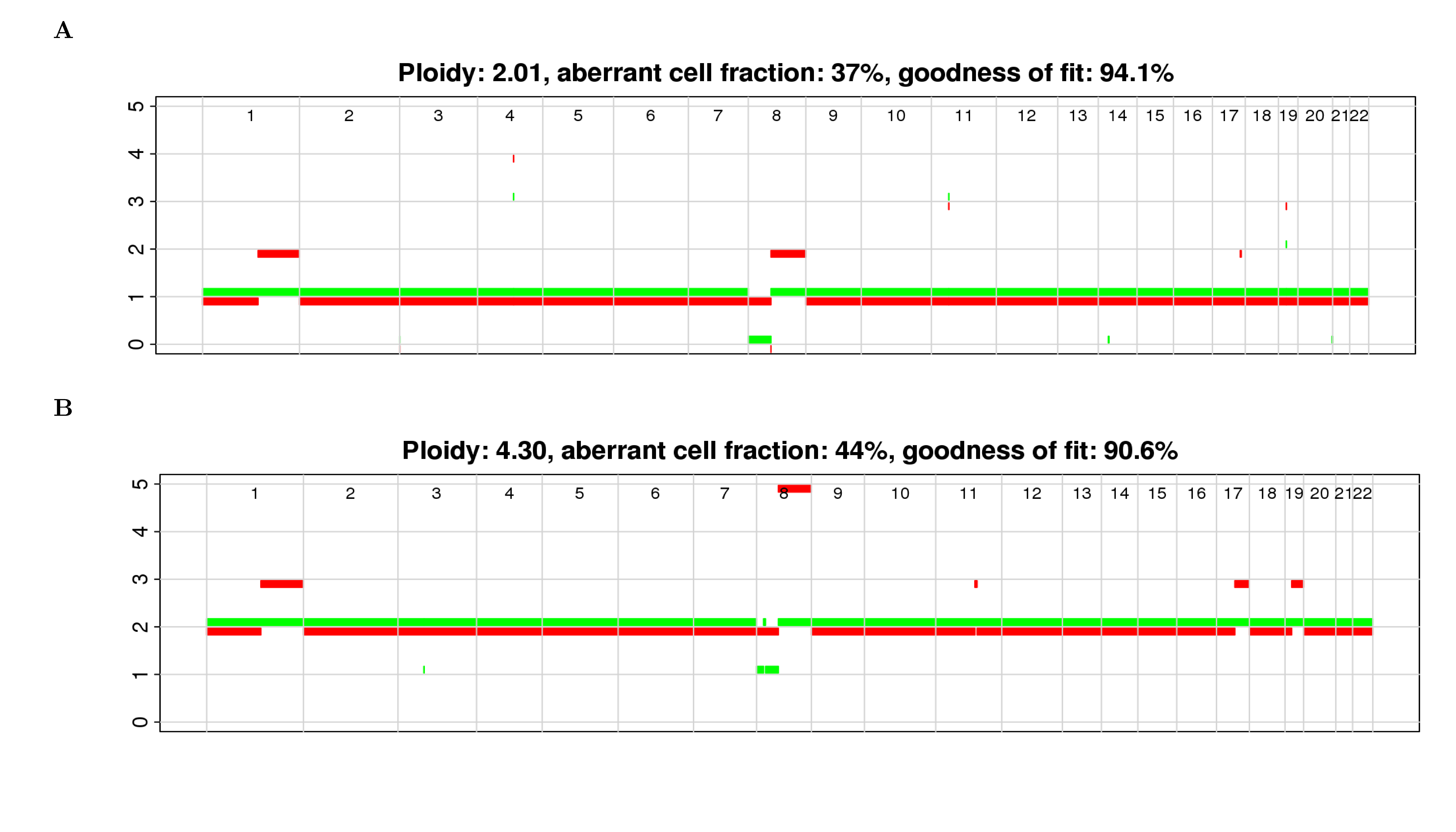

Supplement: S1 Fig — ASCAT profile of two HCCs including a pseudo-diploid sample (A) and a pseudo-tetraploid sample (B) and presenting similar chromosomal aberrations. (TIF) [file pone.0189334.s002.tif]
